# Calcium Disorders in the Emergency Department: Independent Risk Factors for Mortality

**DOI:** 10.1371/journal.pone.0132788

**Published:** 2015-07-14

**Authors:** Thomas C. Sauter, Gregor Lindner, Sufian S. Ahmad, Alexander Benedikt Leichtle, Georg-Martin Fiedler, Aristomenis K. Exadaktylos, Dominik G. Haider

**Affiliations:** 1 Department of Emergency Medicine, Inselspital, University Hospital Bern, Freiburgstrasse, Bern, Switzerland; 2 Centre of Laboratory Medicine, Inselspital, University Hospital Bern, Bern, Switzerland; Kaohsiung Medical University Hospital, TAIWAN

## Abstract

**Background:**

Calcium disorders are common in both intensive care units and in patients with chronic kidney disease and are associated with increased morbidity and mortality. It is unknown whether calcium abnormalities in unselected emergency department admissions have an impact on in-hospital mortality.

**Methods:**

This cross-sectional analysis included all admissions to the Emergency Department at the Inselspital Bern, Switzerland from 2010 to 2011. For hyper- and hypocalcaemic patients with a Mann-Whitney U-test, the differences between subgroups divided by age, length of hospital stay, creatinine, sodium, chloride, phosphate, potassium and magnesium were compared. Associations between calcium disorders and 28-day in-hospital mortality were assessed using the Cox proportional hazard regression model.

**Results:**

8,270 patients with calcium measurements were included in our study. Overall 264 (3.2%) patients died. 150 patients (6.13%) with hypocalcaemia and 7 patients with hypercalcaemia (6.19%) died, in contrast to 104 normocalcaemic patients (1.82%). In univariate analysis, calcium serum levels were associated with sex, mortality and pre-existing diuretic therapy (all p<0.05). In multivariate Cox regression analysis, hypocalcaemia and hypercalcaemia were independent risk factors for mortality (HR 2.00 and HR 1.88, respectively; both p<0.01).

**Conclusion:**

Both hypocalcaemia and hypercalcaemia are associated with increased 28-day in-hospital mortality in unselected emergency department admissions.

## Introduction

Although electrolyte disorders are frequent in emergency departments, there have been few studies of the prevalence and outcome of hypercalcaemia and hypocalcaemia in emergency department populations [1,2].

In contrast, hypocalcaemia is known to be extremely common in the intensive care unit setting (up to 88% prevalence) and correlated with the severity of illness and mortality. No correlation with a specific illness has yet been found [3,4].

In critically ill patients in intensive care units increased mortality is associated with hypocalcaemia, but not hypercalcaemia [4,5]. In most of these patients it was not possible to identify a single cause for the hypocalcaemia [4].

In patients with chronic kidney disease (CKD), a prospective cohort study in Asia found that low serum calcium is an independent prognostic marker of the need for renal transplantation, as well as being a prognostic marker for the renal function of patients with stage 3–4 CKD. In this study, no association was observed between high serum calcium and renal outcome [6].

Case reports suggest that hypocalcaemia is associated with heart failure in both children and adults, perhaps due to a decline in myocardial contractility [7–9]. One study showed that hypocalcaemia is an independent predictive factor for left ventricular diastolic dysfunction in patients with CKD [10].

In patients with acute ST-elevation myocardial infarction, a recent study suggests that serum calcium levels on admission and in-hospital mortality are associated [11].

Moreover, a recent German study suggested that calcium disorders might also be of concern in trauma patients. This study found that hypocalcaemia on the third day after moderate or severe traumatic brain injury is a prognostic factor of early mortality [12].

In the present retrospective study, we have now tested whether abnormal calcium serum levels and in-hospital mortality are associated in unselected emergency department admissions.

## Material, Methods and Patients

This cross-sectional study included all patients admitted to the Emergency Department of the Inselspital between January 1, 2009, and December 31, 2010. Multiple presentations to our department during the study period were excluded and only the first presentation counted. The emergency department of the university hospital of Bern, Inselspital, is treating the whole spectrum of traumatic and non-traumatic emergency patients and is a Level 1 major trauma centre, stroke centre and specialised centre for cardiovascular emergencies. Within this period 22,239 patients were monitored. 8,270 patients with calcium measurement at emergency department admission were included in our study. Patients who had at least one cardiovascular, neurological or metabolic, nephrologic, intestinal or hepatic comorbidity alongside the primary reason for admission received a blood test including calcium concentration. Data from the medical records were collected on age, sex, ethnic group, nationality, outcome, length of hospital stay and blood tests (creatinine, eGFR, sodium, chloride, phosphate, calcium, potassium, magnesium, osmolality). The study protocol for the present cross-sectional study of emergency admissions was approved by the Ethics Committee of the Canton Bern, Switzerland.

In addition, the Ethics Committee of the Canton Bern, Switzerland waived the need for informed consent for the present study due to the retrospective design.

The results are presented as medians and 25–75% interquartile ranges.

For hyper- and hypocalcaemic patients with a Mann-Whitney U-test, the differences between subgroups divided by age, length of hospital stay, creatinine, sodium, chloride, phosphate, potassium and magnesium were compared. With a Pearson`s Chi-Square Test, associations of calcium levels with different parameters (sex, hospitalisation, mortality, ethnic group and diuretic therapy) were tested in univariate analysis.

The effects of different variables on survival were investigated using the Cox proportional hazard regression model. The Kaplan-Meier method was used to compare the 28-day survival and the log-rank test to compare the differences in survival between subgroups. The 28-day mortality was identified through our hospital records.

All calculations were performed with the SPSS Statistics 21 (IBM Coorp.) program. The authors performing the data analysis were not involved in the data acquisition. A two tailed p value of less than 0.05 was considered statistically significant.

Plasma calcium concentrations between 2.20 and 2.55 mmol/l were defined as normal, in accordance with our laboratory reference values.

## Results

**Fig 1:**
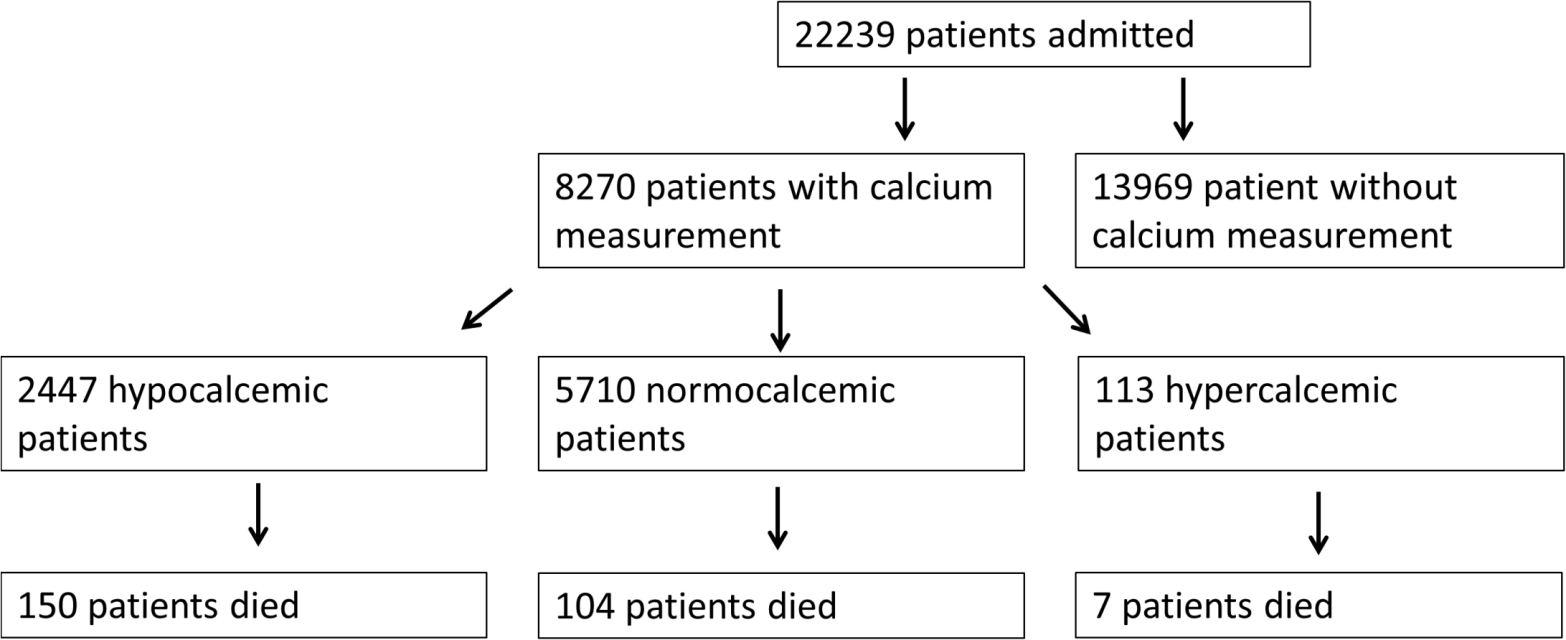
Flowchart. Patient flowchart.

Calcium measurements were performed in the emergency department for 8,270 of 22,239 screened patients (Fig 1).

The baseline characteristics of the 8,270 patients included are shown in Table 1.

**Table 1:** Baseline characteristics of all patients with calcium measurement at hospital admission (n = 8,270).

Parameter	Median (Interquartile range)
Age (years)	58 (41;72)
Length of stay (days)	2 (0;7)
Creatinine (μmol/l)	72 (60;90)
eGFR (MDRD) (ml/min/1,73m^2^)	95 (72;116)
Sodium (mmol/l)	139 (137;141)
Osmolality (mosm/kg)	291 (284;306)
Chloride (mmol/l)	103 (100;106)
Phosphate (mmol/l)	1.01 (0.83;1.19)
Calcium (mmol/l)	2.26 (2.18;2.34)
Potassium (mmol/l)	3.9 (3.7;4.2)
Magnesium (mmol/l)	0.81 (0.73;0.88)

In our cohort, 2,447 patients were hypocalcaemic, 5,710 normocalcaemic and 113 hypercalcaemic.

150 hypocalcaemic patients (6.13%) and 7 hypercalcaemic patients (6.19%) died, in comparison to 104 normocalcaemic patients (1.82%).

Compared with normocalcaemic patients, patients with calcium disorders were markedly older, had to stay longer in hospital and had lower eGFR values (all p<0.05, Table 2). Hypocalcaemic patients also had lower sodium and magnesium levels (p<0.05, Table 2).

**Table 2:** Comparison of patient groups with hypocalcaemia (n = 2,447) and hypercalcaemia (n = 113) with normocalcaemic patients (n = 5,710). Medians and interquartile ranges. (Mann-Whitney U, p<0.05*)

Parameter	Hypocalcaemia	Normocalcaemia	Hypercalcaemia
Age	63 (47;76)*	55 (38;70)	66 (50;75) *
Length of stay	4 (0;10)*	1 (0;6)	3 (0;10) *
Creatinine	83 (68;114) *	71 (60;86)	74 (58;102) *
eGFR (MDRD)	90 (60;117) *	96 (76;116)	72 (47;96) *
Sodium	138 (136;141) *	139 (138;141)	139 (136;141)
Osmolality	292 (283;311)	291 (285;301)	296 (282;312)
Chloride	103 (99;107)	103 (100;106)	100 (94;108)
Phosphate	1.01 (0.82;1.24)	1.00 (0.84;1.16)	1.08 (0.85;1.40)
Calcium	2.12 (2.05;2.16) *	2.31 (2.25;2.37)	2.63 (2.58;2.76) *
Potassium	3.9 (3.6;4.2)	3.9 (3.7;4.2)	4 (3.6;4.3)
Magnesium	0.79 (0.70;0.87)*	0.82 (0.75;0.88)	0.83 (0.73;0.91)

In univariate analysis, calcium levels were associated with sex, mortality and pre-existing diuretic therapy (all p<0.05, Table 3).

**Table 3:** Associations of calcium levels with different parameters in univariate analysis. (Pearson’s Chi-Square Test (p<0.05*)).

Parameter	p-value
Sex	0.002*
Hospitalisation	<0.001*
Mortality	<0.001*
Ethnic group	0.419
Diuretic therapy	<0.001*

Multivariate Cox regression analysis showed that hypocalcaemia and hypercalcaemia were independent risk factors for mortality (all p<0.05, Tables 4 and 5).

**Table 4:** Multivariate Cox regression analysis for 28 day mortality in hypocalcaemic patients (p<0.05*).

Parameter	Hazard ratio (Confidence interval)	p-value
Hypocalcaemia	**2.000 (1.459; 2.741)**	<0.001*
Age	**1.029 (1.019; 1.040)**	<0.001*
Sex	1.045 (0.762; 1.433)	0.783
Creatinine	1.001 (1.000; 1.002)	0.031
Diuretic therapy	0.687 (0.470; 1.003)	0.052

**Table 5:** Multivariate Cox regression analysis for 28 day mortality in hypercalcaemic patients (p<0.05*).

Parameter	Hazard ratio (Confidence interval)	p-value
Hypercalcaemia	**1.881 (1.184; 2.989)**	0.007*
Age	**1.030 (1.014; 1.047)**	<0.001*
Sex	1.004 (0.634; 1.591)	0.986
Creatinine	**1.003 (1.001; 1.004)**	<0.001*
Diuretic therapy	0.883 (0.052; 1.499)	0.645

Age per se is also a risk factor for mortality in these patients (p<0.05, Tables 4 and 5).

Creatinine was only a risk factor for mortality in thepatients with hypercalcaemia (p<0.05, Table 5).

**Fig 2:**
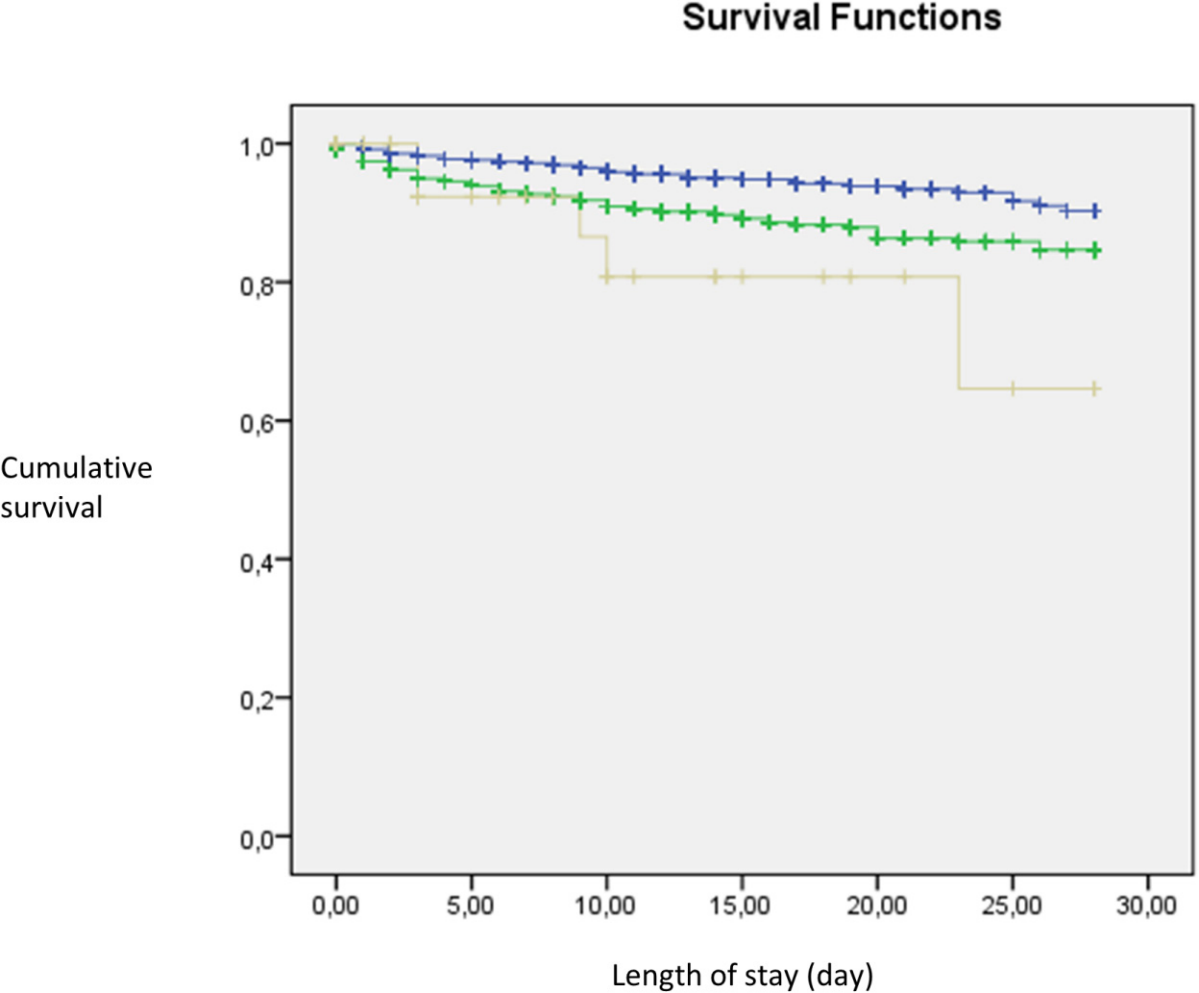
Kaplan Meier plot. Kaplan Meier plot for 28 day in-hospital mortality in patients with normocalcaemia (blue line), hypocalcaemia (green line) and hypercalcaemia (grey line) (Log rank test (p<0.05)).

The Kaplan Meier plot shows the decrease in survival in both patient groups with abnormal calcium levels in comparison with normocalcaemic patients (both p<0.05, Fig 2). The marked decrease can be seen from day 5 (Fig 2).

## Discussion

Our study showed that both hypercalcaemia and hypocalcaemia are independent new risk factors for mortality in emergency departments aside from classical risk factors (creatinine and age). Patients with calcium disorders were older, stayed longer in hospital and had lower eGFR values.

Many factors are known to contribute to increased mortality in chronic and acute kidney disease [13, 14]. Not only end stage kidney disease but also impairment of kidney function have been demonstrated to increase mortality [13]. In our study, creatinine was an independent risk factor for mortality, which is comparable with other study results [15–17]. Overall cardiovascular mortality accounts for nearly half of deaths in patients with chronic kidney disease [9]. As kidney disease progresses, macrovascular disease becomes less important than microvascular problems and electrolyte disorders [18].

The association we found between hypocalcaemia and hyponatraemia and hypomagnesaemia is consistent with other studies [3, 9], for example in intensive care unit patients with sepsis [4].

Hypocalcaemia is often seen after thyroidectomy, common in patients in intensive care units and often of multifactorial aetiology [4]. It has been shown to be a predictor of poor renal outcome and mortality in patients with CKD [4, 5]. Vroonhof et al. found that lower calcium values were associated with increased mortality in unselected emergency department admissions [19]. This increased mortality is confirmed in our study with emergency department patients. In intensive care unit patients, this mortality is not associated with a specific illness but is thought to be part of the severe illness reaction [4]. It is not yet clear whether this accounts for patients admitted to emergency departments. Calcium is necessary for the cardiac myocyte contraction. Whether this is the major cause for the increased mortality remains unclear and needs further research [10].

The increased mortality in hypercalcaemia is consistent with an Asian study on calcium disorders in an emergency department [2]. Hypercalcaemia is often associated with diseases such as cancer [20].

One of the best known causes of hypercalcemia is Parathyroid hormone related peptide (PTHrP) associated hypercalcemia, which is most often found in patients with solid organ malignancy and is associated with poor outcome [21].

In a recent study, the cause of mild hypercalcaemia was only identified in 21% of patients [22]. In addition, only the minority of patients had a follow up or workup plan at discharge [22]. In contrast, the vast majority of these patients, symptoms related to hypercalcaemia could be found [22]. Another study demonstrated that in patients with malignancy nearly half of patients showed symptoms of hypercalcaemia but in none of the medical reports was hypercalcaemia mentioned as the reason for hospitalization [23]. Two Asian studies had higher numbers of hypercalcaemic patients [2, 24]. A possible explanation for this discrepancy might be that calcium was only measured in a third of our population, which might include some selection bias. Due to the retrospective design of our study no additional routine calcium measurements were conducted. This should be topic of further research to exclude selection bias. It appears to be unlikely that additional calcium measurements would have changed the presented results, as general calcium measurements are likely to be within the normal range.

One limitation of our study is a vast heterogeneity of discharge diagnoses. Therefore, associations with endpoints of our study could not be detected. The same limitation exists for the increased mortality shown by Vroonhof et al. for hypocalcaemia [19]. However, registry studies carry that limitation in general. In addition, large discrepancies exist between the initial suspected diagnosis (leading to calcium analysis) and the final discharge diagnosis.

In summary we could show that both hypocalcaemia and hypercalcaemia are associated with increased 28-day in-hospital mortality in unselected emergency department admissions. More attention should be paid to abnormal calcium levels beside classical risk factors and metabolic disorders.
